# Spontaneous intracranial arterial dissection in the young: diagnosis by CT angiography

**DOI:** 10.1186/1471-2377-6-16

**Published:** 2006-04-11

**Authors:** William C Robertson, Curtis A Given

**Affiliations:** 1 Department of Neurology, University of Kentucky, Kentucky Clinic - L445, 740 South Limestone Street, Lexington, Kentucky 40536, USA; 2 Department of Radiology, University of Kentucky, Chandler Medical Center, 800 Rose Street HX311C, Lexington, Kentucky 40536-0293, USA

## Abstract

**Background:**

Spontaneous carotid artery dissections have been rarely reported in children. Diagnosis has traditionally been confirmed by catheter arteriography. More recently diagnosis has been made by magnetic resonance imaging and magnetic resonance angiography; however the sensitivity of these techniques has yet to be determined. The authors are unaware of reports of carotid dissection confirmed by dynamic computed tomography (computerized tomographic arteriography) in the young.

**Case presentation:**

We recently evaluated a fourteen year-old male following the development of transient neurologic symptoms. There was no antecedent illness or trauma. Dynamic computed tomography revealed an intracranial dissection involving the supraclinoid segment of the left internal carotid artery (confirmed by catheter arteriography). Studies for vasculitis, pro-thrombotic states, and defects of collagen were negative.

**Conclusion:**

Spontaneous carotid artery dissection is a potential cause of transient neurological symptoms and ischemic stroke in the pediatric population. Dynamic computed tomography appears to be a reliable diagnostic tool which can lead to early diagnosis.

## Background

Most cases of carotid artery dissection (CAD) in children result from trauma to the oropharynx or neck [1,2]. Spontaneous cervicocranial dissections have been reported in a variety of conditions including Marfan's syndrome, fibromuscular dysplasia, atherosclerosis, luetic arteritis, moyamoya disease, vascular Ehlers-Danlos syndrome (EDS), and cystic medial necrosis [3,4]. Arterial dissections in the absence of systemic disease or antecedent trauma appear to be rare in the young.

In the past diagnosis of arterial dissections has required catheter arteriography. During the last two decades advances in magnetic resonance (MR) imaging and MR angiography (MRA) have improved our ability to diagnose dissection of the cerebral arteries but their reliability remains unknown [5,6].

We recently cared for a fourteen year-old boy who developed transient neurologic signs and symptoms. There was no antecedent history of illness or trauma and no family history of intestinal perforation or early stroke. Initial diagnosis of CAD was made by dynamic computed tomographic scans (CT angio). We are unaware of any reports of CAD confirmed by CT angio in the pediatric age group.

## Case presentation

A healthy fourteen year-old male without antecedent illness or trauma developed bi-temple headaches. Two weeks later he had a one-hour episode of numbness involving the right arm, leg, and face. The spell was associated with slurred speech, difficulty swallowing, and mild expressive dysphasia. A similar but less severe episode occurred four days later. Physical examination was unremarkable. There was no facial or extremity dysmorphism and the skin was normal.

**Figure 1:**
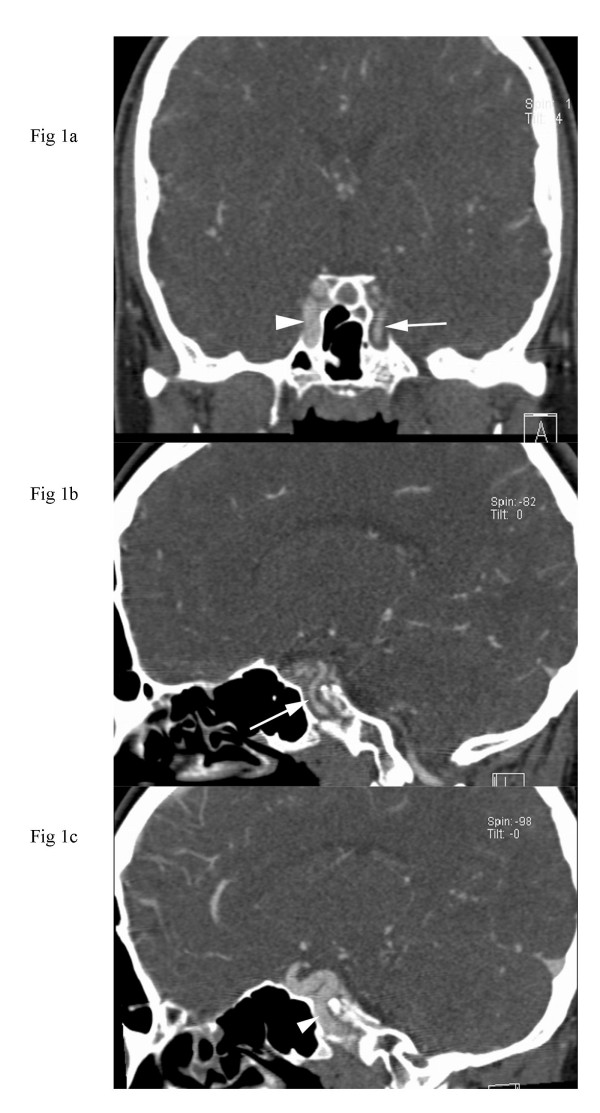
Dynamic computed tomographic scans with maximum intensity projection (MIP) images in the coronal (a) and parasagittal planes (b, c) revealing diminished caliber of the intracranial portions of the left internal carotid artery (arrow). The contralateral right internal carotid artery displays normal caliber (arrowhead).

**Figure 2:**
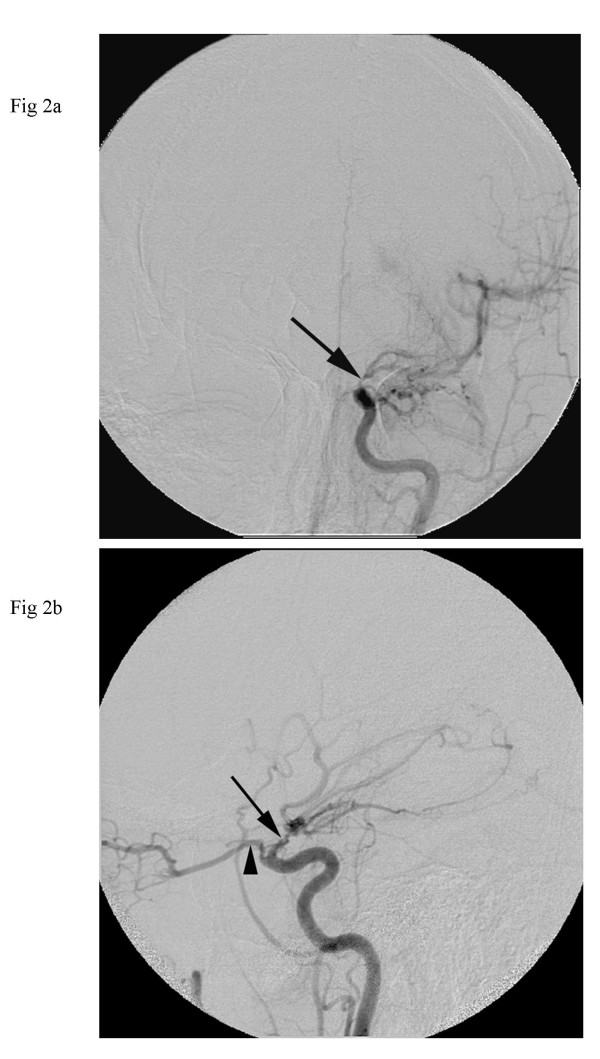
Anterior-posterior (a) and lateral (b) projections from a digital subtraction catheter arteriogram confirm the presence of a dissection (arrow) of the intracranial carotid, just beyond the ophthalmic artery origin (arrowhead).

Carotid Doppler studies revealed intact flow in the common carotid artery (CCA), internal carotid artery (ICA) and external carotid artery. ICA velocities were "blunted" suggesting a distal stenosis. Transcranial duplex examination showed elevated mean velocities in the middle cerebral arteries (MCA's) consistent with a high grade stenosis of either the distal ICA or proximal MCA. CT Angio revealed a dissection of the intracranial portion of the left ICA (Figure 1) which was later confirmed by catheter arteriography (Figure 2). Other studies including tests for vasculitis and prothrombotic states were negative. Echocardiogram was normal. Light and electron microscopy (EM) from a skin biopsy did not reveal ultrastructural abnormalities. Type I and type III procollagen and collagen were examined by protein gel electrophoresis at the Collagen Diagnostic Laboratory of the University of Washington, Seattle, Washington. No abnormalities of synthesis, secretion or electrophoretic mobility of the procollagens were found. Conversion of procollagens to collagen was also normal.

The child was initially treated with intravenous heparin and later with Coumadin. There were no additional symptoms and he was discharged after four days of hospitalization.

## Conclusion

The incidence of CAD in the pediatric population is unknown. Fullerton and co-workers found 118 cases of arterial dissection in patients less than 18 years reported in the English literature from January 1964 through December 2000. The authors concluded that there were 32 spontaneous dissections which were equally divided between the anterior and posterior circulation [5]. The authors did not report how many of these 32 cases were intracranial.

Schievink published the largest single series of spontaneous CAD in children. He described 18 cases, seven of which were intracranial [6]. All of these children were included in Fullerton's article. Since Schievink's report in 1994 we found four additional cases of CAD in patients under age 18 not included in Fullerton's review [1,7-10]. Three of these dissections were intracranial and none had recognized antecedent events.

Our review confirms that cervicocranial arterial dissections rarely occur in children without an antecedent event or underlying systemic condition. We found 36 cases of so-called spontaneous dissection reported in the English literature over the past four decades. We confirmed that at least 10 of these were intracranial and all involved the anterior circulation [1,5-9].

Our patient's presentation of headache followed by signs and symptoms of focal cerebral ischemia is typical of other reported cases of intracranial dissections in the young [6]. All reported children have presented with signs or symptoms of focal cerebral or retinal ischemia; however, similar events are uncommon among adults [5]. Intracranial dissections in children usually involve the anterior circulation while the posterior circulation is more likely to be involved in adults [6]. Fullerton's review suggests that cervicocranial dissections not associated with strenuous activity, trivial trauma, or significant trauma are equally divided between the anterior and posterior circulations [5].

It seems reasonable that cervicocerebral artery dissections in children that occur in the absence of an antecedent event or systemic disease may be secondary to an underlying arteriopathy. Arterial dissection is known to occur in such connective tissue disorders as Ehlers-Danlos and Marfan syndromes. Ehlers-Danlos syndrome (EDS) type IV is one of six recognized forms of EDS and is known to be associated with arterial rupture and dissection. This particular type, now referred to as vascular EDS, results from structural abnormalities of type III collagen. The typical clinical findings include distinctive facial features, thin translucent skin, easy bruising, and rupture of vessels and/or viscera. Less severe defects of type III collagen could result in predisposition to arterial rupture or dissection without full phenotypic expression of the disorder [11].

Several years ago Mayer described multiple spontaneous dissections in a previously healthy thirty-five year old female. There was a history of easy bruisability, but there were no abnormalities on general examination except for bluish sclera. The patient was subsequently found to have a quantitative defect of type I collagen. The authors concluded that some cases of spontaneous arterial dissection could be secondary to unrecognized qualitative and/or quantitative defects of collagen [3].

Brant's 1998 study supports the idea that spontaneous dissections are related to underlying connective tissue abnormalities. He performed light and EM from skin biopsies on 25 patients with spontaneous cervicocerebral dissections. Seventeen cases (68%) had ultrastructural abnormalities resembling those found in EDS type II or type III [11].

We were unable to find any reports of children with spontaneous CAD who have had biochemical assays or molecular biology studies for defective collagen or skin biopsy looking for ultrastructural connective tissue pathology. Attempts to demonstrate abnormal collagen or connective tissue pathology in our patient were unsuccessful. Although systematic studies looking for connective tissue abnormalities have not been done in spontaneous dissections, evidence to date suggests many patients may ultimately prove to have a subtle underlying arteriopathy [3,11].

Diagnosis of cervicocranial arterial dissection has traditionally been made by catheter arteriography. With the advent of MR and MRA diagnosis has been facilitated and more cases are probably being recognized [5]. The typical MR picture of dissection consists of an eccentric signal void (corresponding to the residual lumen) surrounded by a semilunar hyperintensity (secondary to the mural hematoma) on T1 and T2 weighted images [12]. Although the sensitivity of MR is not precisely known, in at least 20% of cases MR will fail to demonstrate typical abnormalities thus potentially delaying treatment [12].

Reports of dynamic CT findings in cervicocranial dissections are uncommon and there are few, if any, reports in the pediatric population. Studies in adults appear to be particularly sensitive for carotid dissections. Zuber and co-workers found CT angiography in adults to be at least as sensitive as MR for detection of arterial dissections. Their report showed dynamic CT to actually be more sensitive than MR in detecting carotid lesions. Abnormalities included eccentric mural thickening in stenotic lesions. The authors also noted instances of a narrowed central or eccentric enhancement from a residual lumen surrounded by hypodensity secondary to a mural hematoma. On occasion the mural hematoma was surrounded by a thin layer of annular contrast [12]. Our patient's study showed a stenotic lesion with eccentric mural thickening as described in some of the adults in Zubers' report.

Our experience and review suggests that spontaneous dissection of the ICA may occur in the young in the absence of systemic disease or readily detectible connective tissue disorders. Zubers' study and our observations suggest that CT angio is at least as reliable as MR in detection of carotid dissections and may lead to earlier diagnosis and treatment. The authors believe patients with characteristic findings on CT angio may not need to be subjected to conventional angiography.

## Abbreviations

1. CAD – carotid artery dissection

2. EDS – Ehlers-Danlos syndrome

3. MR – magnetic resonance imagery

4. MRA – magnetic resonance angiography

5. CT angio – dynamic computed tomographic scans

6. CCA – common carotid artery

7. ICA – internal carotid artery

8. MCA – middle cerebral artery

9. EM – election microscopy

## Pre-publication history

The pre-publication history for this paper can be accessed here:
